# Proteome Unravels Mechanism Differences in Embryogenesis Between Honey Bee Drone and Worker (*Apis mellifera* L.)

**DOI:** 10.1016/j.mcpro.2026.101579

**Published:** 2026-05-05

**Authors:** Beibei Ma, Jianke Li, Hesham R. El-Seedi, Yu Fang

**Affiliations:** 1 State Key Laboratory of Resource Insects, Institute of Apicultural Research, Chinese Academy of Agricultural Sciences, Beijing, China; 2 Pharmacognosy Group, Department of Pharmaceutical Biosciences, Uppsala University, Biomedical Centre, Uppsala, Sweden

**Keywords:** embryogenesis, honey bee, drone, worker

## Abstract

The physiological and social behaviors differ widely between honeybee workers and drones. All the organ rudiments of adult bees are formed during the embryonic stage. The initial molecular bases at the proteomic level for both embryonic developments have been identified, but a comprehensive understanding of the significant events involved in embryonic establishment remains elusive. To elucidate the molecular regulatory mechanisms underlying tissue differentiation during the embryogenesis of drones and workers, we implemented a state-of-the-art approach that combines in-hive inspection and targeted sampling (at nine embryogenesis stages) with high-throughput proteomics technology to investigate the developmental differences. In-hive inspection of hatching timing revealed an average developmental gap of approximately 3.6 h between the two embryos. Furthermore, proteomic analyses indicate that drone and worker embryos adopt distinct developmental strategies. Notably, proteins involved in fatty acid metabolism and key biological pathways related to organ formation—such as the Hedgehog and Wnt signaling pathways—are activated earlier in drones, suggesting that tissue development begins sooner in drone embryos than in workers. Additionally, the upregulation of cytoskeletal proteins and antioxidants in drone embryos likely supports their larger cell size and higher metabolic stress, reflecting distinct molecular characteristics of male development. Ribosomal proteins essential for biosynthetic support remain consistently expressed throughout the late stages in male embryos, indicating that drone embryogenesis lasts longer than that of workers. This work provides novel insights into the molecular foundations of honeybee embryogenesis and lays both theoretical and practical groundwork for future research into the mechanisms driving embryonic development.

## Highlights

• An average developmental gap of approximately 3.6 h between the two embryos.
• Establishment of tissue and organ starts earlier and takes longer in drone embryo.
• Fatty acid pathway lasts longer and antioxidant activates stronger in drone embryogenesis.
• Different patterns of cytoskeleton proteins expressed in drone and worker embryos.

## Graphical Abstract

**Figure:**
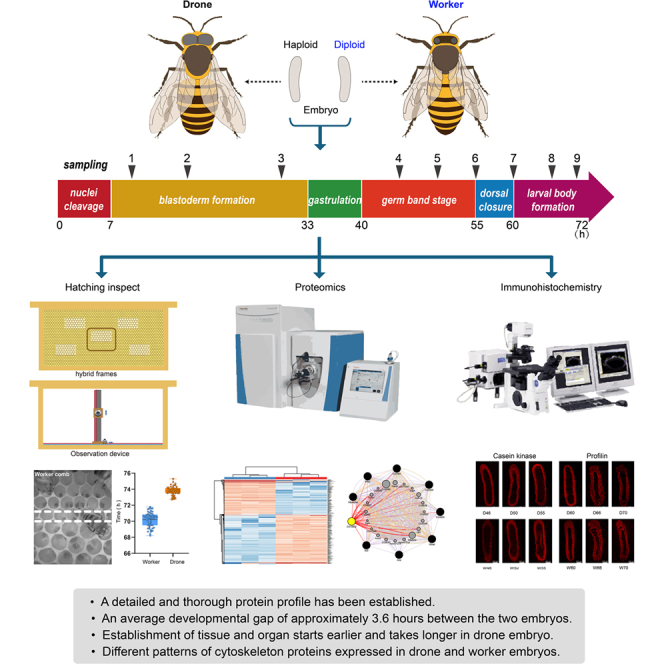

## In Brief

By combining in-hive monitoring with high-throughput proteomics across nine embryonic stages, this work reveals distinct developmental strategies between honeybee drone and worker embryos. Drones activate organogenesis and lipid metabolism pathways earlier, while upregulating cytoskeletal and antioxidant proteins to support larger cell sizes and metabolic stress. Additionally, sustained ribosomal protein expression reflects a prolonged embryonic period in drones. These findings provide a comprehensive molecular blueprint of honeybee embryogenesis, unraveling the regulatory mechanisms that drive male and female phenotypic differentiation.

As eusocial insects, honeybees exhibit a sophisticated division of labor within their colonies and maintain complex social connections among individuals. Each bee fulfills distinct roles and responsibilities: the queen lays eggs, workers maintain colony operations, and drones mate with virgin queens. Although these distinct roles, their developmental processes are quite similar. Honeybees undergo complete metamorphosis through four distinct stages: egg, larva, pupa, and adult. During embryonic development, the formation of various organ rudiments occurs (1), which directly influences the biological characteristics of adult bees. Notably, despite the significant differences in physiological development and social behavior among the drone, worker, and queen, their embryonic development lasts approximately 72 h (1).

To date, research on honeybee embryogenesis has primarily focused on morphological, genetic, transcriptional, and proteomic levels. The morphology and timing of worker embryo development have been elaborated. Generally, the entire process of worker embryogenesis is characterized by four stages: blastoderm formation, during which nuclei undergo mitotic divisions in the periplasm and the dorsal strip forms; gastrulation, characterized by germ layer segregation and pre-serosa formation; germ band formation, when the serosa, amnion, and nervous system develop alongside major organ primordia; and larval body completion, marked by dorsal closure and larval cuticle formation (1). More specifically, biological events such as blastoderm formation, germ layer segregation, and organ system formation have been investigated in depth (2). The incubation of honeybee eggs is influenced by factors such as temperature and genetic background. Temperature variations significantly affect the duration of egg development (3), and the hatching time of eggs from different queens varies considerably, ranging from 66 to 93 h (4).

The development of honeybees has been elucidated through genomic approaches (5). Genomic methods have been applied to study the embryonic development processes of fertilized eggs in diploid worker bees, as well as the coordination and regulation of genome activation in unfertilized eggs of haploid male bees (6). Haploidy and sex determination significantly influence the evolutionary rates of genes within the honeybee genome (7). Furthermore, genome modification serves as an effective means to uncover the molecular biology of various organisms. CRISPR-Cas9 has emerged as a powerful and efficient tool for gene editing in honeybees, which provides a solid foundation for further research into the distinct mechanisms governing male and female bees (8, 9). By utilizing CRISPR-Cas9, genes related to sex determination (10), body color (11), and taste receptors (12) were knocked out successively, which contributes to the basic and applied sciences on honeybees and other insects (13).

Recent transcriptomic analyses have elucidated the complex gene regulation underlying key events in honeybee embryogenesis, such as sex determination and immune responses. Alternative splicing may initiate the sex determination cascade that distinguishes male and female honeybee embryos, representing a more diverse mechanism than previously understood pathways (14). In the early stages of embryogenesis, specific regulatory factors govern the development of haploid and diploid embryos, with early zygotic transcription initiating during the cleavage phase of fertilized eggs (15). Furthermore, the transcriptome of diploid mutant embryos provides essential insights into gene expression differences associated with sex differentiation, which is valuable for further exploring sexual dimorphism and developmental processes in Hymenoptera (9). Additionally, the transcriptomic characterization of honeybee embryonic responses to viral infection contributes to our understanding of transgenerational virus effects (16).

Proteomic approaches have provided new insights and in-depth understanding of honeybee biology (17, 18). Throughout their long evolutionary history, the male embryonic patterns of *Apis cerana* and *Apis mellifera* have developed distinct biological characteristics, which are evident in their respective proteomes (19). Moreover, the morphological development of embryonic drones occurs earlier than that of worker bees and continues into the middle to late stages of development (20). Furthermore, phosphorylation proteomics provides a theoretical foundation for further research on the signaling pathways that regulate embryonic development in honeybees (21). However, high-resolution investigation of embryonic mechanisms requires improved sampling accuracy (shorter time interval) and detailed analysis of the complex events involved in embryonic establishment. Based on our previous findings, this project is designed to provide a comprehensive proteomic landscape of embryonic differentiation by combining higher-resolution proteomics and novel morphological validation, identifying key candidate proteins and pathways that may underlie these regulatory processes.

## Experimental Procedures

### Experimental Design and Statistical Rational

Honeybee colonies were maintained in the apiary of Institute of Apicultural Research, Chinese Academy of Agricultural Sciences in Beijing. All colonies were Italian honeybees (*A. mellifera ligustica*). The queens were all imported from Bologna, Italy, to ensure a stable genetic background. Sampling was conducted in the late spring and summer, a period when nectar sources were abundant and the colonies were healthy, strong, and actively breeding. Three colonies with equivalent strength were selected as the experimental sources (22). Based on our previous findings (20), the sampling points were determined by the corresponding developmental periods, which were not evenly distributed. The queen in each colony was restricted to a specific area for 2 h to lay eggs. Samples were collected at 10, 18, 30, 46, 50, 55, 60, 66, and 70 h after oviposition. For each colony at each time point, totally 1000 worker and 1000 drone eggs were collected respectively. Biological replicates involved three colonies (n = 3). To ensure the sustainability of the sampling colonies, enough new emerging bees as potential nurse labor from the other two reserve colonies were added to compensate for the population shortage. Approximately 100 to 150 eggs were harvested each time, and the collection process was completed within 10 min. This process was repeated until approximately 1000 eggs were accumulated, which constituted one biological replicate for a given time point. Embryonic protein samples were collected from worker-destined eggs (W) and drone-destined eggs (D) at each developmental time point. The primary explanatory variable was worker vs. drone. Worker and drone samples were paired by colony: Wn and Dn (n = 1, 2, 3) originated from the same queen. No technical replicates were performed. Significant differences in protein abundance between drone and worker were determined using the Benjamini–Hochberg false discovery rate (FDR) correction method which implemented in the PEAKS Q quantification module, with an FDR threshold of <0.01 and a fold change threshold of ≥1.5. For Western blotting (WB) and immunohistochemistry (IHC) results, all quantitative data were derived from three independent biological replicates (n = 3) and are presented as the mean ± standard error of the mean. Statistical analyses were performed using GraphPad Prism version 9.0 (GraphPad Software). Unpaired, two-tailed Student’s t-tests were used for comparisons between two groups. One-way analysis of variance (ANOVA) followed by Tukey’s multiple comparisons test was used for comparisons of more than two groups, *p*-value <0.05 was considered statistically significant.

### Chemical Reagents and Honeybee Colonies

Unless otherwise stated, all chemicals were purchased from Sigma-Aldrich.

### Hatching Observation

**Fig. 1:**
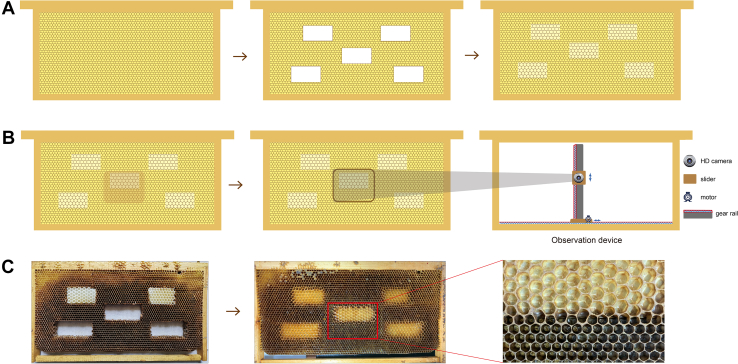
**The hybrid frame for in-hive inspection.***A*, the process of replacing the worker frame bars with drone combs. Five wax bars representing different positions were removed from a complete worker frame. Then the gaps were replaced by drone combs with same shape. The modified frame was put into hive until bees fix it with wax completely. *B*, the queen was forced to lay eggs in each hybrid position of the frame for 1 hour with a queen excluder. The process of hatching was recorded using an in-hive camera from 65 h after oviposition. *C*, physical hybrid frame and the inspection area.

To detect the developmental gap of embryogenesis between drones and workers, hybrid frames containing both male and female cells were modified. Specifically, five rectangular comb blocks were removed from drone frames using an electric strip heater and then stuffed into the corresponding hollowed areas of the worker frame. The hybrid frames were then placed in the hive until the blocks were fused by secreted beeswax (Fig. 1*A*). Finally, a queen was confined to the fusion comb for 1 hour to allow egg-laying. Approximately 66 h later, a high-definition camera was installed inside the bee colony to observe the hatching process in real-time (Fig. 1, *B* and *C*). The mixed comb areas were divided into grids, and the duration from egg to C-shaped larva was recorded. Statistical analyses of hatching time were performed using Descriptives in IBM SPSS, version 25 for Windows (IBM Corp). Meanwhile, to obtain samples at late stages of embryonic development for further morphological analysis, drone and worker eggs at 66 and 70 h of development were fixed in a 1:1 mixture of n-hexane and 4% paraformaldehyde.

### LC-MS/MS Analysis of Protein

#### Protein Preparation

Protein extraction was performed following our established methods (23). Frozen embryo samples were homogenized using a lysis buffer composed of dimethylammonio-1-propanesulfonate, 30 mM dithiothreitol, and 20 mM Tris-base. The homogenate was then centrifuged at 12,000*g* for 15 min at 4 °C, and the supernatant was carefully transferred into a new tube. Ice-cold acetone was then added to precipitate the proteins, and the mixture was incubated at −20 °C for 30 min before being centrifuged again at 12,000 g for 10 min at 4 °C to pellet the proteins. The final precipitate was dissolved in 5 M urea. Protein concentrations were determined by the Bradford assay (24). Denatured proteins were reduced with dithiothreitol and alkylated with iodoacetamide to prevent the reformation of disulfide bonds (25). Prior to enzymatic digestion, the protein solution was diluted with 50 mM ammonium bicarbonate buffer (pH 8.0) containing 10 mM glycine to ensure the final urea concentration was reduced to 0.5 M or less. Subsequently, the samples were digested using sequencing-grade modified trypsin (Promega) and incubated for 12 to 14 h at 37 °C. The digestion reaction was terminated with formic acid (FA), and desalination was conducted with C18 solid-phase extraction cartridges (CDS Analytical LLC). Finally, the peptides were pooled and dried using a Speed-Vac system (RVC 2–18, Martin Christ, Germany) in preparation for MS/MS analysis.

#### MS Analysis

The digested peptides were resuspended in 0.1% FA prior to mass spectrometry (MS) analysis. The solution was then centrifuged at 12,000*g* for 20 min at 4 °C, and the supernatant was carefully transferred to a new tube. The concentration of the peptide solution was measured by a protein quantification spectrophotometer (Nanodrop 2000, Thermo Fisher Scientific), and the sample concentration was adjusted to 0.25 μg/μl with 0.1% FA. Then 8 μl of peptide solution was analyzed using a Thermo Orbitrap QE-HF coupled with an Easy-nLC 1200 system. Briefly, peptides were loaded onto an Easy-Spray trap column packed with 2 μm C18 (100 Å, 100 μm × 2 cm, Thermo Fisher Scientific) and subsequently separated on an analytical column packed with 3 μm C18 (100 Å, 75 μm × 15 cm, Thermo Fisher Scientific). The flow rate for the liquid chromatography samples was maintained at 350 nl/min, while the sample loading rate was set at 5 μl/min. Mobile phase A consisted of 0.1% FA, and mobile phase B comprised 80% acetonitrile with 0.1% FA. The elution process lasted for a total of 120 min, following this gradient: from 3% to 8% buffer B over the first 5 min; from 8% to 20% buffer B over the next 8 min; from 20% to 30% buffer B over a period of 20 min; from 30% to 90% buffer B within another span of 5 min; and finally maintaining at a constant concentration of 90% buffer B for an additional 10 min. For ionization, we employed electrospray ionization with a mass range of m/z = 350-1550 and a resolution setting of 120,000. Spectra were acquired in higher energy collisional dissociation mode using highly pure nitrogen as the collisional gas, with normalized collision energy set at 28%, isolation window fixed at 2.0 m/z, and scan resolution established at 15,000. The top 20 MS/MS scans were collected.

#### Protein Identification

The raw mass spectrometry data were imported into Peaks Studio software (version 8.5, Bioinformatics Solutions Inc.) for analysis, utilizing the *A. mellifera* protein database (NCBI, April 2023, 21778 entities) and the cRAP protein database (NCBI, April 2022, 115 entities) (21). The search parameters were established as follows: precursor and fragment mass tolerances were set to 20 ppm and 0.05 Da, respectively. The enzyme specificity was defined as trypsin, allowing for a maximum of two missed cleavages. A fixed modification of carbamidomethyl (C, +57.02) and a variable modification of oxidation (M, +15.99) were applied. The maximum number of allowed variable post-translational modifications per peptide was set to three. The false discovery rate (FDR) was maintained at less than 1% at both the peptide and protein levels. Only proteins identified with at least two unique peptides were considered valid. The MS proteomics data have been deposited in the ProteomeXchange Consortium (http://proteomecentral.proteomexchange.org) *via* the iProX partner repository (26, 27), with the dataset identifier PXD037018.

#### Label-Free Quantitation of Protein Abundance

To quantify the changes in protein abundance between drone and worker embryos at nine developmental points, a label-free strategy was employed using Peaks Studio 8.5 (Bioinformatics Solutions Inc.). The mass error tolerance was set to 20 ppm, while the retention time shift tolerance was established at 6.0 min.

Protein quantification was based exclusively on unique peptides, with protein abundance calculated as the sum of extracted ion chromatogram peak areas from unique supporting peptides. The expression level of each protein was determined by the median of peptide ratios across the three replicates. Statistical significance was determined using the Benjamini–Hochberg FDR correction method implemented in the PEAKS Q quantification module, with an FDR threshold of <0.01 and a fold change threshold of ≥1.5. Normalization was applied to calibrate data between different sample runs and to correct for systematic experimental variations encountered during sample analysis.

#### Bioinformatics Analysis

To gain an in-depth understanding of the biological functions of proteins in drones and worker embryos, the functional annotation and enrichment analysis of the differentially expressed proteins were performed using the ClueGO (28) plugin (version 2.5.8) within Cytoscape (version 3.8.2, INSERM, AVENIR Team, Integrative Cancer Immunology). The analysis was based on the *A. mellifera* Gene Ontology (GO) database. Enrichment for GO terms in the Biological Process category was calculated using a two-sided hypergeometric test. To control for multiple testing errors, the resulting *p*-values were adjusted using the Bonferroni step down method, and only terms with a corrected *p*-value <0.05 were considered significantly enriched. For functional grouping and network visualization, a Kappa score threshold of 0.4 was used.

To identify the biological pathways associated with the differentially expressed proteins, KEGG (Kyoto Encyclopedia of Genes and Genomes) pathway enrichment analysis was performed using the KOBAS 3.0 web server (http://kobas.cbi.pku.edu.cn/) (29). The amino acid sequences (in FASTA format) of the differentially expressed proteins, exported from PEAKS Studio, were submitted to the KOBAS platform. *A. mellifera* (honey bee) was selected as the species in mapping parameter settings. The statistical significance of the enrichment for each pathway was determined using a hypergeometric test. To control the multiple testing, the resulting *p*-values were subsequently adjusted using the Benjamini-Hochberg procedure to calculate the False Discovery Rate (FDR, q-value). Pathways with a q-value <0.05 were considered to be significantly enriched.

To further explore potential functional connections among the identified proteins, we constructed protein–protein interaction (PPI) networks using GeneMANIA within Cytoscape. This tool leverages an extensive array of functional association data, including both protein and genetic interactions (30). We integrated known and predicted PPI datasets from the *Drosophila melanogaster* genome into GeneMANIA for analysis.

### Verification

#### Western Blotting (WB)

To validate the quantitative results, WB was performed according to previously described methods (31). Briefly, 10 μg of protein samples were separated using stacking (4%) and separating (12%) sodium dodecyl sulfate-polyacrylamide gel electrophoresis (SDS-PAGE), with each sample run in triplicate. The proteins were transferred from the gel to PVDF membranes using an iBlot Gel Transfer System (Invitrogen) at 20 V for 7 min. The membranes were blocked in TBST containing 5% skimmed milk for 1 hour and then incubated overnight at 4 °C with anti-casein kinase (1:1000), anti-acetyl-CoA acetyltransferase (1:2000), and anti-profilin antibodies (1:1000). Anti-glyceraldehyde 3-phosphate dehydrogenase antibody (1:5000) served as the reference control. Commercially available primary antibodies were sourced from Abcam. Following three washes with TBST, the membranes were incubated with HRP-conjugated secondary antibodies (1:5000). Protein bands were visualized using chemiluminescence, and imaging was conducted with E-BLOT (e-BLOT Life Science). Quantification of the bands was performed using ImageJ software (National Institutes of Health). Specifically, the integrated density of each target protein band was measured after background subtraction. The relative expression of each target protein was normalized to the corresponding internal control (GAPDH) to account for variations in protein loading. The final results were expressed as the mean ± standard deviation from three independent experiments.

#### Immunohistochemistry (IHC)

To verify the expression trends of key regulators (casein kinase and profilin) at the histological level, immunohistochemical staining was performed. Freshly collected embryos were fixed in a 4% paraformaldehyde solution for 24 h. Routine histopathological profiles were conducted using the tissue processing system (Shandon Citadel 2000, Thermo Fisher Scientific). The samples were then embedded in paraffin and sectioned to a thickness of 5 μm using a rotary microtome (Leica RM 2255). The sections were placed onto lysine-coated slides and baked in an oven at 70 °C for 1 hour to ensure proper adhesion. Rehydration of the slides was achieved through a series of solutions: xylene (40 min), 100% ethanol (6 min), 95% ethanol (2 min), 90% ethanol (2 min), 80% ethanol (2 min), 70% ethanol (2 min), and distilled water (5 min). Antigen retrieval was performed by boiling the slides in 0.01 M citrate buffer for 10 min. Following antigen retrieval, the slides were blocked with 2% bovine serum albumin (BSA) in PBS for 1 hour at room temperature. Subsequently, the sections were incubated overnight at 4 °C with primary antibodies diluted at a ratio of 1:200 in a humidified chamber. Finally, secondary fluorescent antibodies labeled with Cy5 were applied to the sections and incubated at room temperature for 1 hour. Fluorescence imaging was acquired using a confocal microscope (FV1000, OLYMPUS), and fluorescence intensity within regions of interest in the images was quantified using ImageJ software (National Institutes of Health) (32). For WB and IHC results, all quantitative data were derived from three independent biological replicates (n = 3) and are presented as the mean ± standard error of the mean. Statistical analyses were performed using GraphPad Prism version 9.0 (GraphPad Software). Unpaired, two-tailed Student’s t-tests were used for comparisons between two groups. One-way analysis of variance (ANOVA) followed by Tukey’s multiple comparisons test was used for comparisons of more than two groups, *p*-value <0.05 was considered statistically significant.

#### Morphological Analysis

To reveal the internal structure of honeybee embryos, we fixed the embryo for morphological analysis. Briefly, the eggs, treated with fixative solution for 24 h, were transferred by an end-modified pipette tip to phosphate-buffered saline (PBS) for rinsing. Then the eggs were transferred onto a glass slide. The images were captured with a stereomicroscope (Eclipse TS100, Nikon).

## Results and Discussion

**Fig. 3:**
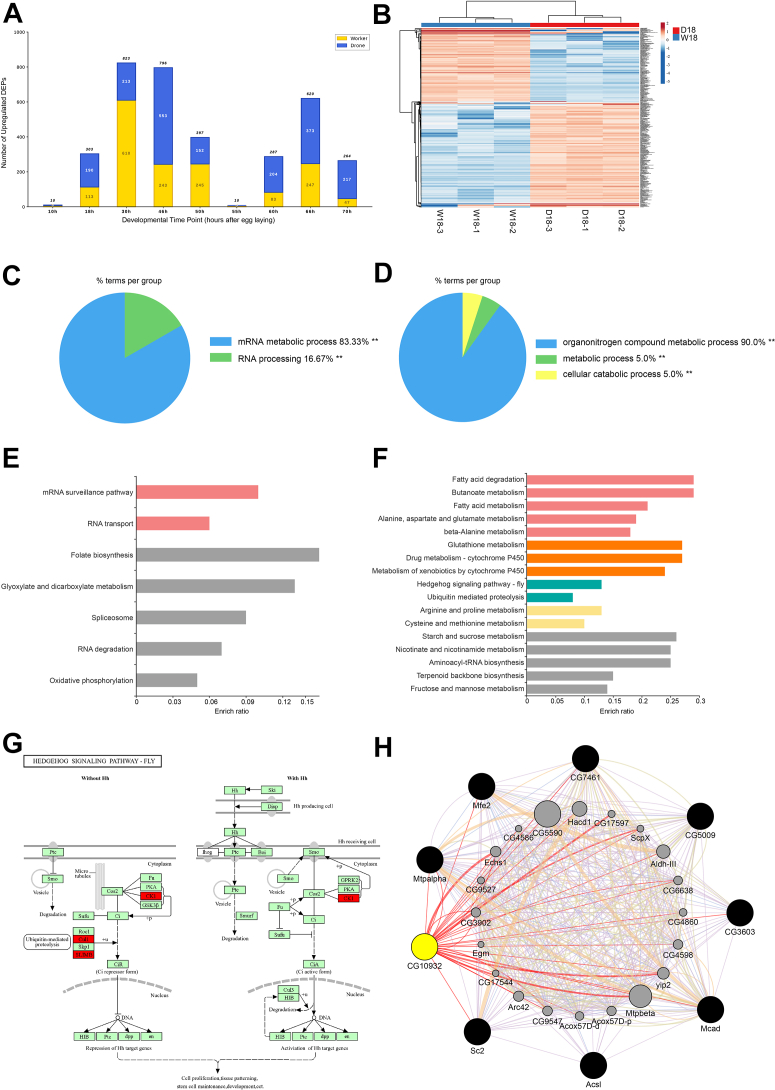
**Quantitative analysis of the differentially expressed protein during the embryonic development of drone and worker at 18 h.***A*, figure A shows the number of proteins identified in worker and drone bees at nine developmental time points (10 h, 18 h, 30 h, 46 h, 50 h, 55 h, 60 h, 66 h, and 70 h after egg laying). The grouped bar chart displays the quantitative results of protein identification (qualitative proteomics) for worker and drone. Worker bees are represented in *yellow*, while drone bees are represented in *blue*. *B*, the heat map was based on 303 quantified embryos proteins. Cluster analysis uses an online software (https://biit.cs.ut.ee/clustvis/). *C*, biological functions of up-regulated proteins in the embryos of honeybee workers at 18 h. The functional gene ontology (GO) categories were annotated using ClueGO (version 2.5.8) within Cytoscape (version 3.8.2). The significantly enriched functional gene ontology categories in biological processes were determined by comparing the input data with the background of gene ontology annotations in the honeybee genome using a right sided hypergeometric test. The nodes in functionally grouped networks were connected based on a kappa score of 0.4. The use of single and double asterisks signifies significant enrichment at the 0.05 and 0.01 levels of statistical significance, respectively. *D*, biological functions of up-regulated proteins in the embryos of honeybee drones at 18 h. *E*, biological pathway enrichment of up-regulated proteins in the embryos of honeybee workers at 18 h. Significantly enriched pathways were analyzed by KEGG Orthology-Based Annotation System (KOBAS, http://kobas.cbi.pku.edu.cn) (*p* < 0.05). The pathway enrichment was conducted by a hypergeometric statistic test. *F*, biological pathway enrichment of up-regulated proteins in the embryos of honeybee drones at 18 h. *G*, hedgehog signaling pathway map enriched by upregulation of protein expression in 18 h drones. The Representative Enriched Pathway Map was sourced from KOBAS. The *green-labeled boxes* represent protein references of honey bee annotated to the KEGG PATHWAY database, while the *highlighted red* ones signify the protein entries that have been mapped to significantly enriched pathways. *H*, PPI network diagram of proteins in the fatty acid metabolism pathway enriched by upregulated protein expression in 18 h male bees. CG10932 represented acetyl CoA acetyl transferase (ACAT). The visualizations of the interactions between the upregulated proteins in the enriched pathways was rendered using the Genemania plugin within Cytoscape.

**Fig. 4:**
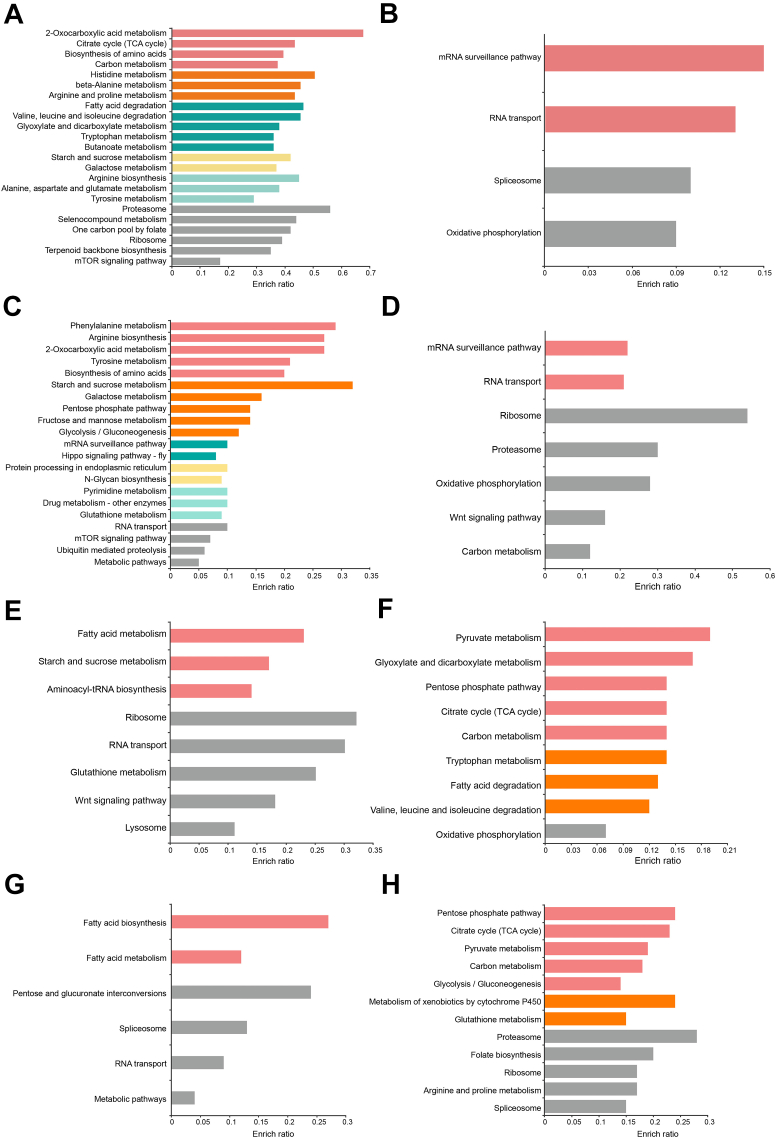
**Biological pathway enrichment.***A*, biological pathway enrichment of up-regulated proteins in the embryos of honeybee workers relative to drones at 30 h. *B*, biological pathway enrichment of up-regulated proteins in the embryos of honeybee drones relative to workers at 30 h. *C*, biological pathway enrichment of up-regulated proteins in the embryos of honeybee workers relative to drones at 46 h. *D*, biological pathway enrichment of up-regulated proteins in the embryos of honeybee drones relative to workers at 46 h. *E*, biological pathway enrichment of upregulated proteins in the embryos of honeybee workers relative to drones at 50 h. *F*, biological pathway enrichment of upregulated proteins in the embryos of honeybee drones relative to workers at 50 h. *G*, biological pathway enrichment of upregulated proteins in the embryos of honeybee workers relative to drones at 66 h. *H*, biological pathway enrichment of upregulated proteins in the embryos of honeybee drones relative to workers at 66 h.

To investigate the molecular differences in embryonic development between drones and worker bees, a comprehensive proteomics approach was employed. A total of 2471 protein groups were identified across the embryonic development of the honeybee (Supplemental Table S1), spanning from 10 to 70 h. The specific numbers of proteins identified in drones and worker bees at each time point, as well as the number of differentially expressed proteins (Supplemental Tables S2, S4, S6, S8, S10, S12, and S14), are presented in and Figure 3*A* and Supplemental Fig. S1, respectively. To decipher the biological changes during embryonic development comprehensively, bioinformatics approaches were applied. Furthermore, GO and KEGG analyses were performed to select the key functional categories and pathways (Figs. 3, 4, Supplemental Figs. S2, S3, Supplemental Tables S3, S5, S7, S9, S11, S13, and S15). Overall, discernible differences in the molecular processes of embryonic development were observed between drones and worker bees. Specifically, in comparison to worker bees, proteins associated with tissue and organ development were expressed for a longer duration in male bees. Ribosomal proteins that provide essential substances for embryonic development, along with proteins related to fatty acid metabolism that support this process and antioxidant proteins that maintain cellular homeostasis, displayed distinct expression patterns in the embryos of worker bees and male bees. Additionally, the expression patterns of cytoskeletal proteins varied between the two groups. Drone embryonic development begins earlier and concludes later at the molecular level, extending a total of 4 hours longer than that of worker bees. Our proteomic analysis reveals a coordinated, sex-specific molecular regulatory program in honeybee embryonic development. Rather than isolated molecular differences, the temporal divergence in drone and worker embryogenesis reflects asynchronous activation of multiple molecular processes at different developmental stages. This work provides novel insights into the molecular mechanisms underlying honeybee embryonic development, establishing a theoretical and practical foundation for further exploration into these developmental processes.

### Asynchronous Embryogenesis Between Haploid and Diploid Honeybees

**Fig. 2:**
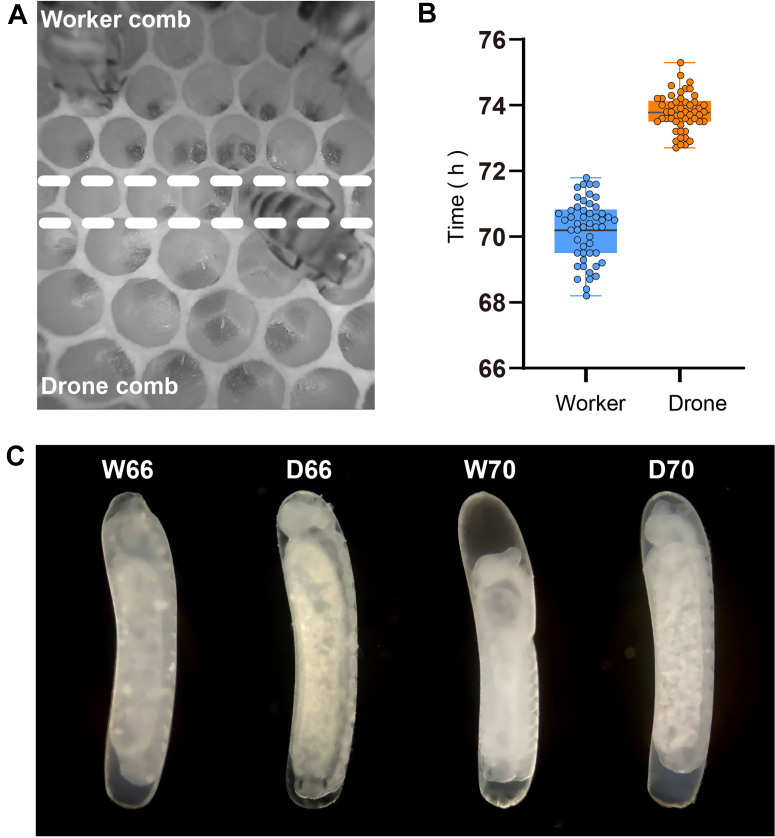
**The developmental gap between drone and worker embryos by in-hive inspection, and morphological analysis.***A*, the drone and worker cells were separated by double-dashed line. The drone embryos in lower larger cells were still in the embryonic stage, while the worker larvae in upper smaller cells had already hatched out (Supplemental Video). *B*, comparison of hatching duration between drone and worker according to the inspection recording by the high-definition camera. the results showed that an average developmental gap of 3.6 h between the two embryos. *C*, internal morphology of worker and drone embryos at 66 and 70 h. The letters D and W represent drones and worker, respectively.

Previous studies have found that the development time of male eggs is about 3 h longer than that of female eggs (33). In contrast, we explored a novel approach by using a fused frame to ensure the synchronized laying of different eggs by the queen. Furthermore, to minimize the measurement error in developmental duration by ensuring a precise start time, the queen's egg-laying was limited to a 1-h window. Besides, to observe the hatching embryos more precisely and dynamically, an in-hive recording device was developed. Based on the improvement above, we detected the average hatching time of embryos (worker 70.2 h, drone 73.8 h, Fig. 2*B*). Therefore, a temporal gap between the embryonic development of drones and workers was discovered (Fig. 2, *A* and *B* and Supplemental Table S16), with drones having a longer development time. Furthermore, the development process of worker bee eggs is faster based on the internal morphological analysis. Within 70 h, the worker bee eggs have fully differentiated and taken on a larval form, and are about to hatch, while the male bee eggs are still undergoing final preparation (Fig. 2*C*). The incubation time of honeybee eggs is co-regulated by multiple factors. Temperature is a primary determinant; for example, worker eggs reared at 34.8 °C hatch approximately 1.4 h faster than those reared at 34.3 °C (3). Additionally, genetic background influences the rate of development. Significant variations in hatching time (66–93 h) exist among eggs from different queens, with European and Africanized honey bees showing average incubation times of approximately 73.3 h and 69.9 h, respectively (4). Our study controlled these variables by maintaining stable colony temperatures and sourcing worker and drone eggs from the same queen for each replicate. Despite these controls, we still observed a significant difference in incubation times. This result indicates that the divergence in developmental progression between worker and drone eggs in our study is primarily due to their intrinsic biological characteristics, rather than external environmental conditions or genetic background.

During oviposition, the queen selectively controls fertilization. As a chorionated egg passes from the lateral oviducts into the common oviduct, it moves past the opening of the spermathecal duct. For diploid (female) eggs, a muscle at the base of the duct known as Breslau's sperm pump deposits a small number of spermatozoa onto the egg's micropyle. The valve fold, located opposite the spermathecal duct, aids this process by pressing the egg against the duct's opening. Unfertilized (haploid) eggs, which develop into drones, simply pass through without receiving sperm. This fertilization event is the critical distinction that initiates either female or male development (34), which leads to the subsequent sperm penetration and successful fertilization. Therefore, worker embryo undergoes an additional fusion step compared to haploid one. Furthermore, the actual karyogamy of the male and female pronuclei during the queen’s laying of a diploid fertilized egg takes approximately 93 ± 7.3 min (35), which might reflect in a phenotype of late initiation in embryogenesis of fertilized eggs. While haploid drone embryos skip the sperm-egg fusion, which serves as a primary factor contributing to their triggering earlier. This temporal delay in developmental initiation has profound consequences for the entire developmental program. The sperm-egg fusion serves as a trigger of differential development temporally. In drone embryos, which bypass sperm-egg fusion, the early developmental pathways initiate at 18 h. In contrast, worker embryos experience a ∼93-min pause (32) to conduct sperm accession and infusion. The temporal gap is reflected in proteomic results. Drones activate early developmental pathways (*e.g.*, fatty acid metabolism) at 18 h (Fig. 3*F*), while workers still have not activated them at this stage. The differences are not obvious probably at the initial stage, but the magnified effects appear in the middle stage. The biological pathway, Wnt signaling, is launched at a time interval of 4 h approximately (drone 46 h, worker 50 h, Fig. 4, *D* and *E*, Supplemental Tables S7, and S9). The cumulative effect of the initial molecular delay, propagated through developmental cascades, is consistent with the systematic temporal asynchrony observed across multiple molecular processes in the proteomic data.

### Sustained Launching of Fatty Acid Metabolism Features Drone Embryogenesis

**Fig. 6:**
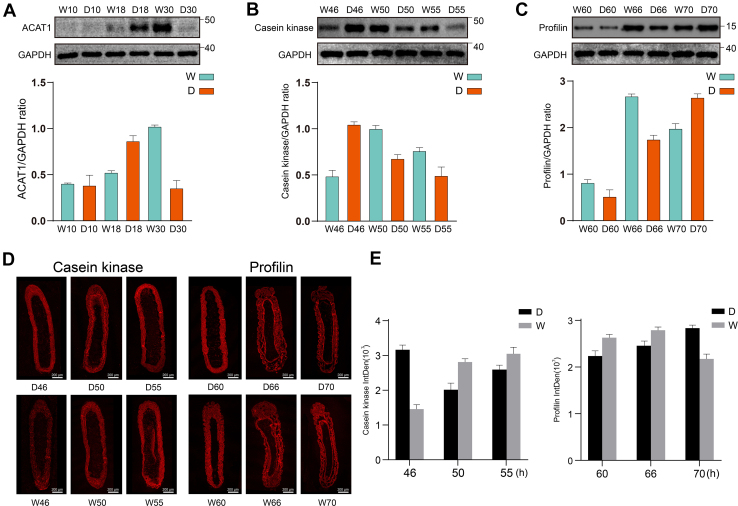
**Western** b**lotting (WB) and Immunohistochemistry (IHC) validation results.***A–C*, the WB showed the relative expression trend of acetyl CoA acetyl transferase (ACAT), Casein kinase and Profilin in the honey bee (*A.m.ligustica*) worker and drone at the three stages (normalized by GAPDH). The error bar was standard deviation. *D* and *E*, the expression intensity of Casein kinase and Profilin in the honeybee embryo. The IHC showed that the expression levels of Casein kinase were different in worker bee and male bee embryos at 46, 50, and 55 h. And the expression levels of Profilin were different in worker bee and male bee embryos at 60, 66, and 70 h. Scale bar, 200 μm.

Fatty acid metabolism and degradation pathways are essential for honey bee embryonic development, as lipids stored in the yolk serve as the primary source of energy and building materials (36, 37). These fatty acids play a crucial role in nutrient accumulation and the formation of cellular membranes and organs during this developmental stage (36). At the onset of embryonic development (18 h), 303 proteins exhibited differential expression between drone and worker bees (Supplemental Table S2 and Fig. 3, *A* and *B*). Notably, proteins upregulated in drone embryo, such as peroxisomal multifunctional enzyme type 2 and acetyl CoA acetyl transferase (ACAT), were enriched in the fatty acid metabolism pathway significantly (*p* < 0.05) (Supplemental Table S3 and Fig. 3*F*). The peroxisomal multifunctional protein-2 is required for the degradation of 2-methyl-branched fatty acids, very long chain fatty acids, and bile acid intermediates in mice (38), suggesting this protein may be providing the necessary energy to drive honeybee embryonic development. As the initial enzyme in the cholesterol synthesis pathway, acetyl-CoA acetyltransferase is involved in the biosynthesis and degradation of ketone bodies in rat tissues (39, 40). Ketone bodies serve as an alternative metabolic fuel source, playing multi-dimensional roles in fuel metabolism, signaling, and cellular homeostasis (41). ACAT may supply both materials and energy for embryonic growth. Furthermore, ACAT was identified as a key protein in the fatty acid metabolism pathway and was selected for validation through PPI analysis (Fig. 3*H*). The elevated expression of ACAT in drone embryos at 18 h, as confirmed by Western blot analysis (Fig. 6*A*), suggests that drones are utilizing lipid metabolism to provide energy for cell division.

Similar results were present in worker embryos at 30 h. Among the 823 proteins that were differentially expressed between drone and worker embryo (Supplemental Table S4 and Supplemental Fig. S1*C*), those upregulated in workers were significantly enriched in the fatty acid degradation pathway (Fig. 4*A* and Supplemental Table S5), including the mitochondrial trifunctional enzyme subunit (Supplemental Table S4). This enzyme plays a crucial role in lipid mobilization and energy production through fatty acid beta-oxidation in mice (42), indicating that it may provide a substantial amount of energy for worker bee embryo development during this stage.

In the middle to late stages of honeybee embryogenesis, the presence of fatty acid metabolism-related proteins and biological pathways reflects the intensity of rudiment establishment. Fatty acid metabolism and fatty acid degradation pathways were significantly enriched in worker embryos at 50 h and in drone embryos at 60 h (Supplemental Tables S9, S11, Figs. 4*E*, and Supplemental Fig. S3*B*), respectively. Furthermore, key proteins that act as central regulators of lipid metabolism, such as fatty acid synthase (FAS) and enoyl-CoA hydratase (ECH), in addition to its role in energy storage through fatty acid biosynthesis, FAS is also involved in the synthesis of membrane components necessary for cell division, protein modification, cell signaling, and cell proliferation (43). Fatty Acid Synthase plays a critical role in intestinal adenoma formation and colorectal cancer (CRC) initiation in the Apc-driven mouse model of CRC (44). This suggests that FAS may be associated with intestinal formation during the middle to late stages of embryogenesis. ECH promotes the proliferation and migration of colorectal and gastric cancer cells through the Akt/GSK3β signaling pathway (45, 46), indicating it may play a potential role in the development of organs such as the midgut and honey sac in bees. From an energy support perspective, the trends in the activation of fatty acid-related pathways reflect the fact that drone embryogenesis begins earlier and concludes later than that of worker embryos.

### Earlier Organogenesis in Drone Embryos

The establishment of organ rudiments is a top priority during honeybee embryonic development. The activated biological pathways related to tissue and organ formation support the construction of embryonic organs, including the Hedgehog, mTOR, Hippo, and wnt signaling pathways. The Hedgehog signaling pathway is evolutionarily conserved and plays a crucial role in cell proliferation and differentiation, tissue homeostasis, and tissue patterning during embryonic development (47, 48, 49, 50). In 18-h embryos, the upregulated proteins expressed by drones were significantly enriched in the Hedgehog pathway (*p* < 0.05, Supplemental Table S3 and Fig. 3*F*), indicating that events of embryonic morphogenesis are actively occurring at this stage. Specifically, related proteins such as beta-TrCP and casein kinase I were identified (Supplemental Tables S2, S3, and Fig. 3*G*). β-TrCP is critical for cell cycle progression and migration (51) and is involved in blastoderm formation during mouse embryogenesis (52). While casein kinase I is associated with processes such as chromosome segregation, cell cycle progression, and cytokinesis in zebrafish development (53, 54).

**Fig. 5:**
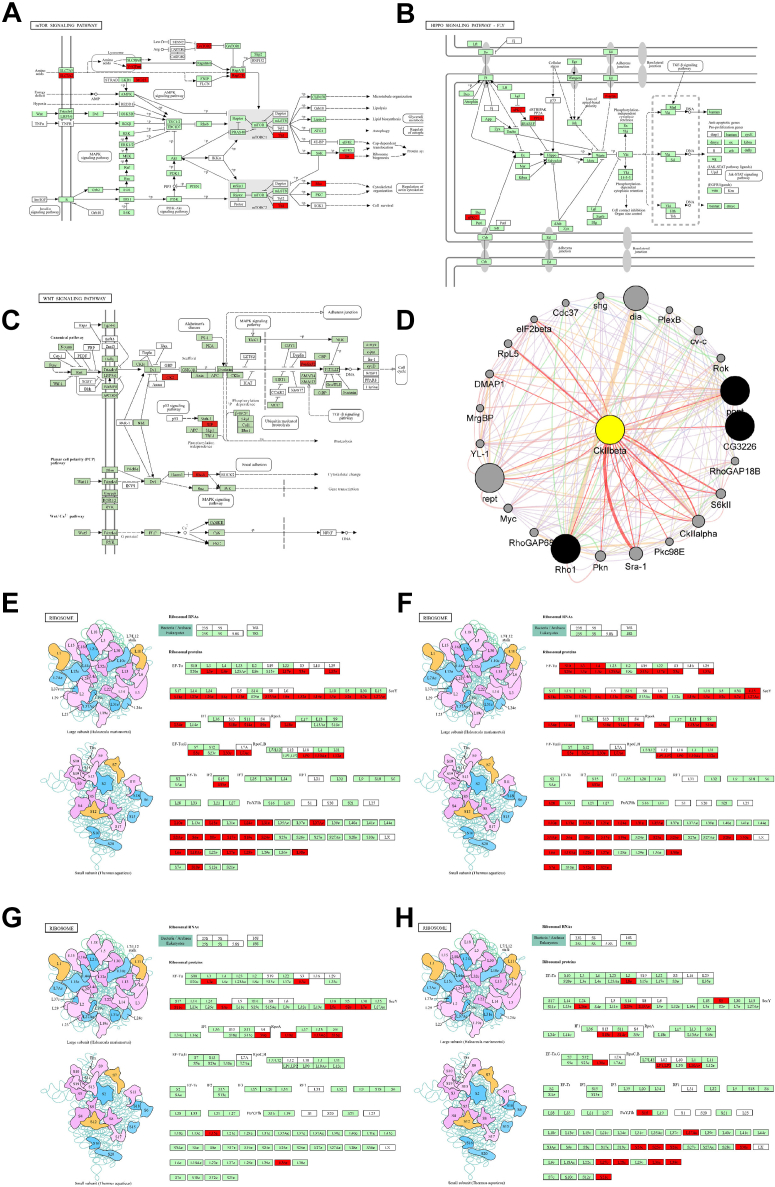
**The Representative Enriched Pathway Map.***A*, the mTOR signaling pathway map was enriched within the KEGG pathway of upregulated protein expression in worker bees at 30 h. *B*, Hippo signaling pathway map was enriched within the KEGG pathway of upregulated protein expression in worker bees at 46 h. *C*, Wnt signaling pathway map was enriched within the KEGG pathway of upregulated protein expression in drone bees at 46 h (*D*) PPI network diagram of proteins in the fatty acid metabolism pathway enriched by upregulated protein expression in 46 h drones. CKIIbeta represents Casein kinase. The visualizations of the interactions between the upregulated proteins in the enriched pathways was rendered using the Genemania plugin within Cytoscape. *E*, Ribosome map was enriched within the KEGG pathway of upregulated protein expression in worker bees at 30 h. *F*, Ribosome map was enriched within the KEGG pathway of upregulated protein expression in drone bees at 46 h. *G*, Ribosome map was enriched within the KEGG pathway of upregulated protein expression in worker bees at 50 h. *H*, Ribosome map was enriched within the KEGG pathway of upregulated protein expression in drone bees at 66 h.

Furthermore, the mTOR signaling pathway takes charge of integrating various environmental signals to control cell cycle progression, cellular proliferation, and growth (55, 56). Worker embryos at 30 h exhibited activation of the mTOR pathway (Supplemental Table S5 and Fig. 4*A*), indicating that proliferation processes were occurring in the worker embryonic cells, in which proteins SEC13 and nucleoporin SEH1 were involved (Supplemental Table S4 and Fig. 5*A*). SEC13 is a dual-function protein with roles in both COPII-mediated protein trafficking from the endoplasmic reticulum to the Golgi apparatus and in nuclear pore complex (NPC) function. Loss of SEC13 function impairs protein trafficking, leading to digestive system organogenesis defects in zebrafish (57), while loss of NPC function of Sec13 impairs nucleo-cytoplasmic transport, resulting in retinal developmental defects (58). Meanwhile, nucleoporin SEH1 is essential for murine brain development (59) and plays a crucial role in female germline function during *Drosophila* oogenesis (60).

The Hippo pathway is another crucial regulator of organ and tissue development, maintaining the balance between cell proliferation and apoptosis to control organ size during development (61, 62). Worker bees activated the Hippo signaling pathway at 46 h (Supplemental Table S7, Figs. 4*C*, and 5*B*), which may be linked to the regulation of body size growth in worker bees. For example, atypical protein kinase C is enriched in this pathway and plays a vital role in the normal development of *Drosophila* epithelial cells and neuroblasts (63, 64), as well as in the formation of multiple lumens in the zebrafish (65).

In addition to the pathways mentioned above, Wnt signaling pathways play a pivotal role in embryonic development, regulating essential physiological processes such as cell differentiation, fate determination, and repair (66, 67, 68, 69, 70). The Wnt signaling pathway has been linked to heart development and repair, demonstrating its critical involvement in the proliferation and differentiation of progenitor cells into cardiomyocytes, as well as in the development of cardiovascular and neural tissues (71, 72). The Wnt pathway was activated in the embryos of drones and workers at different developmental stages, indicating that the progression of rudimentary formation is not synchronized. The 46-h drone embryo exhibited activation of the Wnt signaling pathway (Supplemental Table S7 and Fig. 4*D*), with upregulated expression of proteins such as casein kinase II (CK2), calcyclin, GTP-binding protein Rho1, and ruvB-like 1 (Supplemental Tables S6, S7, and Fig. 5*C*). As a highly conserved protein kinase in all eukaryotes (73), casein kinase II is involved in various cellular processes, including DNA repair, cell cycle regulation, and circadian rhythm control (74, 75); CK2 may be related to intestinal formation in honey bee embryogenesis (32). Calcyclin is highly expressed in proliferating and differentiating epithelial cells in mouse and rat, suggesting its involvement in epithelial cell formation and differentiation (76). In *Saccharomyces cerevisiae*, Rho1, a homolog of mammalian RhoA, is essential for vegetative growth and regulates cell morphology and cytokinesis through its localization at growth sites (77). The small GTP-binding protein Rho1 is a multifunctional regulator of the actin cytoskeleton that is essential for cell viability and cell polarity, and is involved in actin patch localization, septum formation, and cell wall synthesis in the fission yeast *Schizosaccharomyces pombe* (78), which may be linked to the cytoskeletal formation in honeybee embryonic cells. Meanwhile, ruvB-like protein plays a pivotal role in directing neuroectodermal differentiation of mouse embryonic stem cells through chromatin remodeling-mediated gene expression regulation (79).

The evidence above indicates that the drone embryo begins to develop organ rudiments at 46 h, such as neuroectoderm, while the worker embryo activates this pathway slightly later (Fig. 4*E*). At 50 h, the worker embryo showed elevated expression of related proteins, including glycogen synthase kinase 3β (GSK3) and casein kinase II (Supplemental Tables S8, and S9). GSK3 is a multifunctional serine/threonine (S/T) kinase present in all eukaryotic cells, acting in crucial roles in cell proliferation, differentiation, protein synthesis, and apoptosis (80, 81) (Fig. 5*D*). Furthermore, the expression trends of casein kinase at 46- and 50-h time points during embryogenesis were confirmed by Western blotting (Fig. 6*B*), along with quantitative results from immunofluorescence analysis (Fig. 6*D*). These findings suggest that tissue and organ differentiation in male bees occurs earlier during the mid-stages of embryonic development.

### Delayed Ribosome Activation Supports Extended Embryonic Period in Drone

Ribosomes play a fundamental role in protein synthesis and proliferation during bee embryonic development, thereby providing the essential materials for these processes. During the embryogenesis of worker bees at 30 h, 47 proteins were significantly enriched in the ribosome pathway (*p* < 0.05, Supplemental Table S5 and Fig. 4*A*), including ribosomal protein L17 (RPL17) (Supplemental Table S4 and Fig. 5*E*). As a component of the 60S subunit involved in protein synthesis, RPL17 is critical for facilitating ribosome biogenesis (82) and may be related to the rudimentary formation of muscle tissue during honeybee embryo development. The upregulated expression of ribosome-related proteins at this early stage (30 h) of worker bee embryonic development likely provides sufficient resources for proper growth and differentiation.

The ribosomal pathway was activated in 46-h male embryos, with the upregulated expression of 67 proteins (Supplemental Table S7 and Fig. 4*D*), including the 40S ribosomal protein S3 (Fig. 5*F*). Ribosomal proteins exhibit varying expression levels across different tissues and function in a context-dependent manner during animal development. For instance, the depletion of RpS3 in male fly germline cells results in severe defects in spermatogenesis and fertility (83). Therefore, the expression of RpS3 in drone embryos at 46 h may be associated with the formation of male reproductive organs during this embryonic stage.

Coincidentally, drones and workers adopt different strategies in the ribosomal pathway during the late stages of embryonic development. In worker embryos, the pathway was significantly (*p* < 0.05) enriched at 50 h (Supplemental Table S9, Figs. 4*E*, and 5*G*). In contrast, the drone embryo activated this pathway at 66 h (Supplemental Table S13, Figs. 4*H*, and 5*H*). These results indicate that both embryos are enhancing tissue and organ rudiments in preparation for hatching; however, drone embryogenesis takes slightly more time, which was consistent with the embryonic results of observations (Fig. 2, and Supplemental Video). Furthermore, the upregulated protein in drone embryos at 66 h is the 60S ribosomal protein L8, which plays a crucial role in regulating cell size across multiple tissues during *Drosophila* development (84). The high expression of ribosome-related proteins during the late stages of drone embryonic development (66, 70 h) may reflect an increased demand for materials necessary for growth and body size expansion, requiring additional proteins to support this phase of development.

### Stage-Specific Cytoskeletal Protein Expression Reflects Distinct Developmental Strategies in Drones and Workers

Cytoskeletal proteins are closely related to the processes of cell division, cytoskeletal support, and the formation of tissues and organs. During embryonic development, they provide a dynamic scaffold that is essential for tissue establishment. Both drone and worker bees exhibit elevated expression of specific cytoskeletal proteins during the early stages (18 h) of embryonic development. In drones, actin-interacting protein 1 (AIP1) is upregulated. In human and mouse intestinal epithelial cells, AIP1 controls epithelial junction assembly and permeability and regulates actin dynamics in multiple mammalian tissues (85, 86), which may be involved in the initial steps of honeybee epithelial morphogenesis. However, worker embryos increased the expression of tubulin protein at 18 h (Supplemental Table S2), which facilitates the cephalic mesoderm during maximal germband extension in *Drosophila* (87). Therefore, the results suggest that similar biological events could occur during the early development of honeybee embryos.

Drones and workers activated the expression of cytoskeleton-related proteins for different purposes during the middle stages of embryogenesis. Drones promoted the expression of cytoskeletal proteins to form rudimentary tissues. For example, F-actin capping protein was upregulated at 30 h in drone embryos (Supplemental Table S4), as a regulator in Hippo signaling pathway, which regulates *Drosophila* tissue and organ growth (88, 89). Additionally, the actin-depolymerizing factor homology (ADF-H) is upregulated in 46-h drone embryos (Supplemental Table S6). In human T cells, the ADF-H domain protein COTL1 regulates actin dynamics at the immune synapse by antagonizing cofilin, promoting lamellipodial protrusion (90, 91). ADF-H domain proteins more broadly regulate actin filament dynamics at multiple cellular locations through diverse mechanisms, contributing to the formation of contractile and protrusive structures (90, 91). In contrast, worker embryos enhanced the expression of cytoskeletal proteins to establish intercellular junctions and networks. Actin-related proteins (Arps) showed elevated expression at 30 h in worker embryos (Supplemental Table S4); they modulate actin assembly and are components of large protein complexes. Arps are essential for cortical actin cytoskeleton organization during polarized cell growth through interactions with profilin in the fission yeast *S. pombe* (92, 93). This may be linked to the assembly of actin during embryonic development, providing the necessary force for cell movement. Actin-related protein 3 (ARP3) was upregulated in 46-h worker embryos (Supplemental Table S6). The Arp2/3 complex, of which ARP3 is a component, generates branched actin networks essential for cell motility, vesicular trafficking, cytokinesis, and intercellular junction formation. Elevated ARP3 expression in ulcerative colitis promotes apoptosis of intestinal epithelial cells in humans and mice (94, 95). This process may be associated with the formation of cavity structures in organ rudiments of honeybee embryo.

Dorsal closure is a critical biological event that occurs at the end of honeybee embryogenesis (31). Profilin, a biomarker for the dorsal cord closure process, is essential for normal cell division and morphology (96, 97). Quantitative results show that profilin was highly expressed in 66-h worker (Supplemental Table S12) and 70-h drone embryos, which is further validated by Western blotting and immunohistochemistry (Fig. 6, *C*–*E*). This direct evidence demonstrates that dorsal cord closure in drone embryos occurs later than in worker embryos, which is in line with the phenotypic findings from hatching observations (Fig. 2).

### Reactivation of Antioxidant Process in Drone Embryo

Like other living organisms, honeybee embryos have developed a complex network of antioxidants to prevent oxidative damage and mitigate the effects of reactive oxygen species generated by intense metabolic activity (20). During the middle stage (30 h) of development, worker embryos enter a period of active metabolism, during which antioxidant proteins, such as superoxide dismutase 2 (SOD2) and elongation factor Tu (EF-Tu) are significantly upregulated (Supplemental Table S4, *p* < 0.05). SOD2 helps maintain the cellular redox state by protecting cells and tissues from the accumulation of reactive oxygen species (98, 99). EF-Tu plays a crucial protective role in preventing oxidative damage to the postburn myocardium in Sprague-Dawley rats (100). The elevated expression of EF-Tu at this stage (30 h) may be associated with the elimination of free oxygen radicals during the heart formation process in worker bees. The drone embryo initiates the antioxidant process twice during its development. At 46 h, peroxiredoxin-5 and superoxide dismutase 1 (SOD1) were upregulated (Supplemental Table S6). Prx5 promotes cancer invasion in human OSCC cells and mice, supports cell survival in human osteoarthritic chondrocytes *via* Wnt/β-catenin signaling (101, 102), and Prx members in the mud crab *Scylla paramamosain* protect cells from oxidative damage by catalyzing peroxide reduction (103, 104). Superoxide dismutase 1 (SOD1) is an antioxidant enzyme that eliminates free radicals by catalyzing the conversion of superoxide radicals to hydrogen peroxide and oxygen (105, 106, 107, 108, 109). SOD1 also regulates cellular processes during neural tube morphogenesis in chick embryos (106).

At 66 h, the drone embryo activated the antioxidant function once again by increasing the expression of thioredoxin peroxidase 3 (TPx3) and superoxide dismutase 2 (Supplemental Table S12). In *A. cerana cerana*, TPx3 maintains redox homeostasis, protects organisms from toxic reactive oxygen species accumulation, resists paraquat-induced oxidative stress, and safeguards DNA from oxidative damage (110, 111). The primary function of antioxidants is to protect cellular components that are vulnerable to damage from free radicals. Furthermore, the elevated expression of these antioxidants coincides with the peak time of biological metabolism, providing indirect evidence to support the differences in developmental timing between drone and worker embryos.

## Conclusion

We investigated the molecular regulatory mechanisms of tissue differentiation during the embryogenesis of honeybee bee drone and worker through in-hive inspection and high-resolution proteomics, spanning nine developmental periods during embryogenesis. Notably, we found the establishment of tissue and organs starts earlier and takes longer in the drone embryo. Specifically, a developmental gap of about 3.6 h between the embryogenesis of the drone and the worker. Furthermore, the fatty acid pathway lasts longer, and antioxidant systems activate more strongly in drone embryogenesis, along with the distinct patterns of cytoskeleton protein expression. The results enhance our understanding of the molecular basis of bee embryogenesis and provide both theoretical and practical foundations for further research into the mechanisms of embryonic development.

## Data Availability

The MS proteomics data have been deposited in the ProteomeXchange Consortium (http://proteomecentral.proteomexchange.org) *via* the iProX partner repository, detailed sample metadata, including colony identity, sample, developmental time point, and biological replicate number, are provided in the project description on iProX (PXD037018).

## Supplementary data

This article contains supplemental data. https://drive.google.com/file/d/1a-22SrTK2MRU9GJ_6SIzlI9WLNLZw3s0/view?usp=drive_link

## Conflict of interest

The authors declare that they have no conflicts of interest with the contents of this article.
