# Supplementary material for: Proteome Unravels Mechanism Differences in Embryogenesis Between Honey Bee Drone and Worker (Apis mellifera L.)
Source: Mol Cell Proteomics. 2026 May 5;25(6):101579. doi: 10.1016/j.mcpro.2026.101579 (PMC13240821; doi:10.1016/j.mcpro.2026.101579)
Supplement: Supplementary Figures and legends-2 [file mmc2.docx]

**Figure_S1**


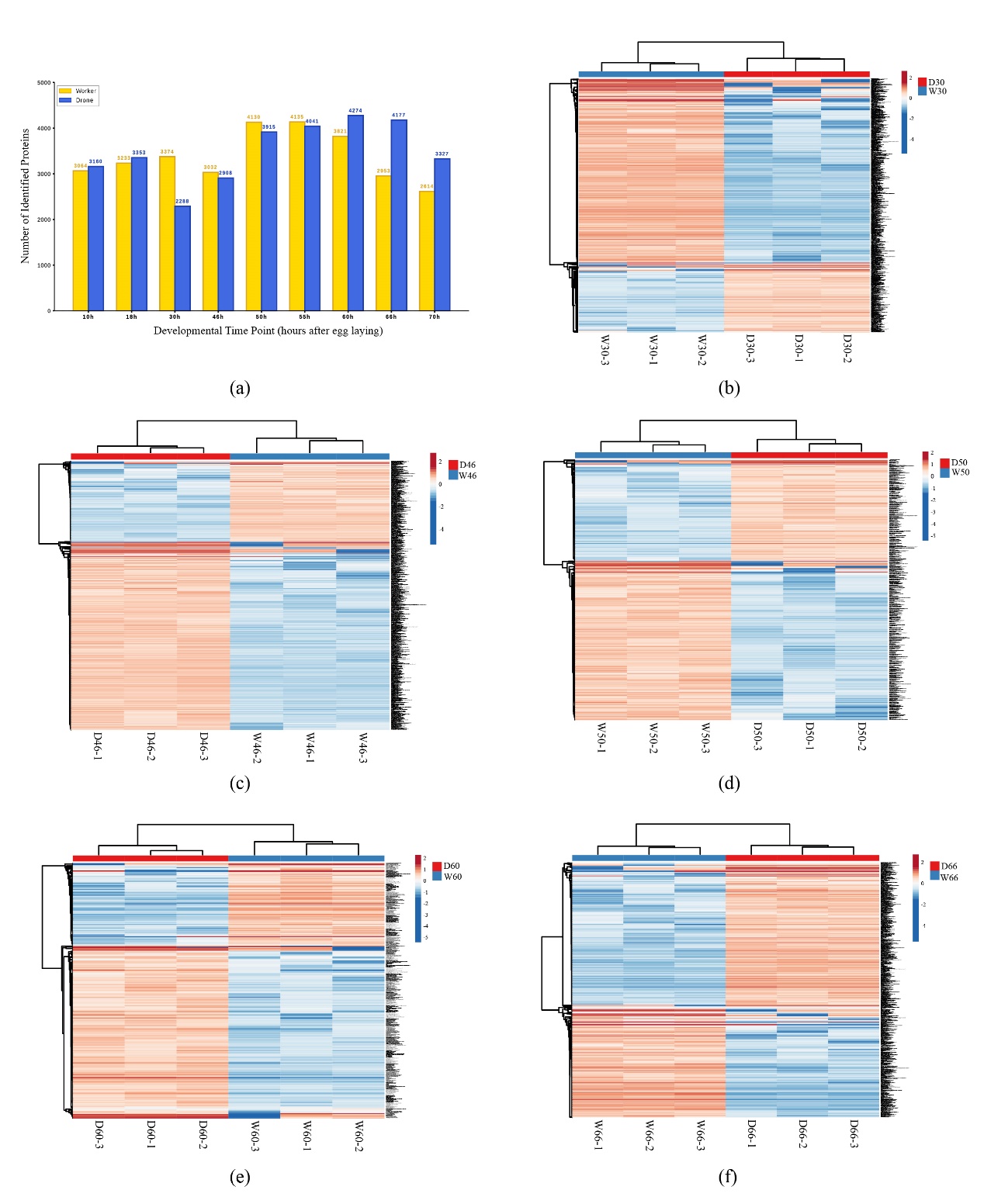


**Figure_S1 Protein identification and clustering analysis of differentially expressed proteins.**

(A) The number of proteins identified in worker and drone embryo at nine developmental time points (10h, 18h, 30h, 46h, 50h, 55h, 60h, 66h, and 70h after egg laying). (B) The heat map was based on 823 quantified proteins from 30-hour embryos. Cluster analysis uses an online software (<https://biit.cs.ut.ee/clustvis/>). (C) The heat map was based on 796 quantified proteins from 46-hour embryos. (D) The heat map was based on 397 quantified proteins from 50-hour embryos. (E) The heat map was based on 287 quantified proteins from 60-hour embryos. (F) The heat map was based on 620 quantified proteins from 66-hour embryos.

**Figure_S2**


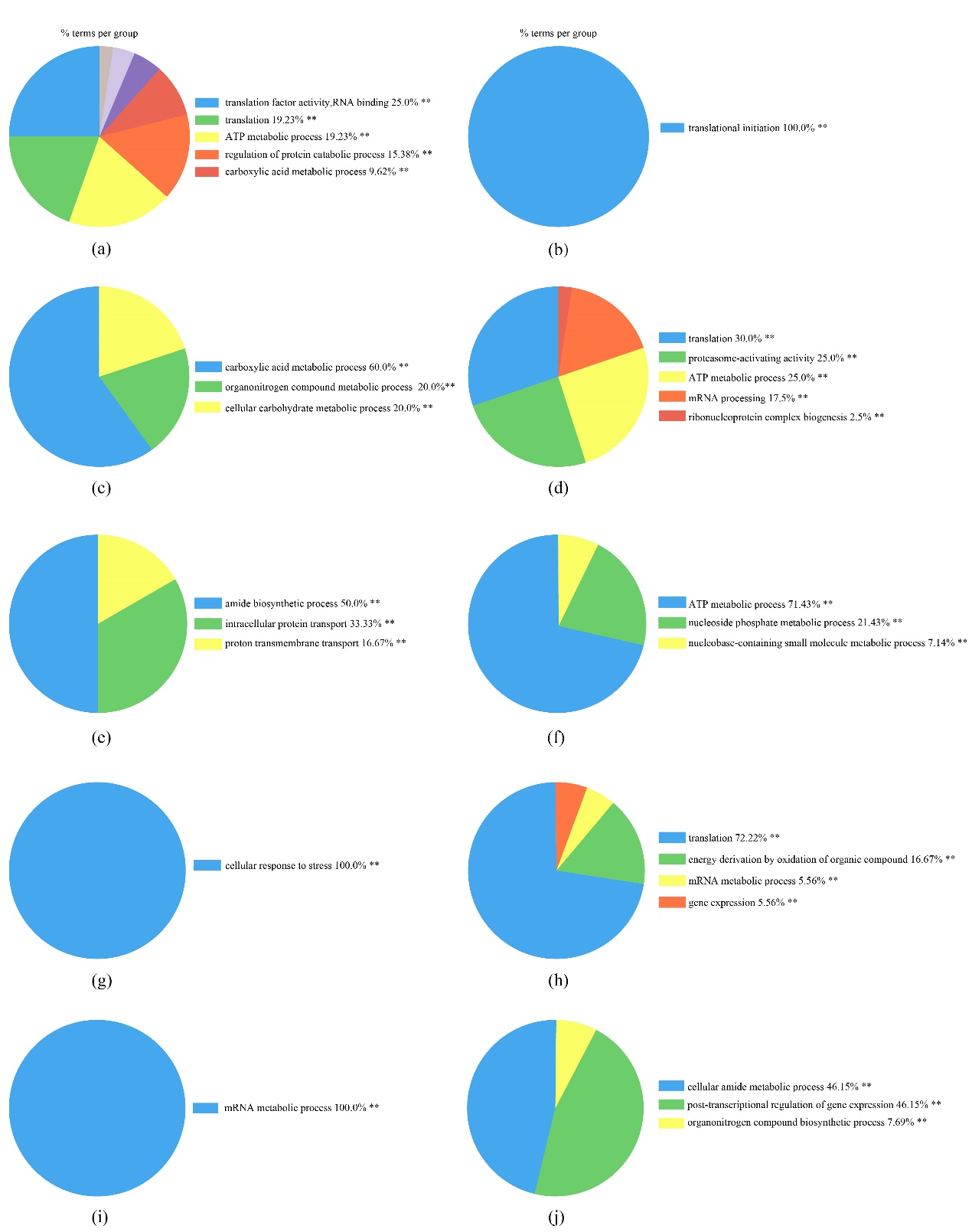


**Figure_S2 GO categories of the differentially expressed protein during the embryonic development of drone and worker.**

(a) Biological functions of up-regulated proteins in the embryos of honeybee workers relative to drones at 30 hour. The functional gene ontology (GO) categories were annotated using ClueGO (version 2.5.8) within Cytoscape (version 3.8.2). The signiﬁcantly enriched functional gene ontology categories in biological processes were determined by comparing the input data with the background of gene ontology annotations in the honeybee genome using a right sided hypergeometric test. The nodes in functionally grouped networks were connected based on a kappa score of 0.4. The use of single and double asterisks signiﬁes signiﬁcant enrichment at the 0.05 and 0.01 levels of statistical signiﬁcance, respectively. (b) Biological functions of up-regulated proteins in the embryos of honeybee drones relative to workers at 30 hour. (c) Biological functions of up-regulated proteins in the embryos of honeybee workers relative to drones at 46 hour. (d) Biological functions of up-regulated proteins in the embryos of honeybee drones relative to workers at 46 hour. (e) Biological functions of up-regulated proteins in the embryos of honeybee workers relative to drones at 50 hour. (f) Biological functions of up-regulated proteins in the embryos of honeybee drones relative to workers at 50 hour. (g) Biological functions of up-regulated proteins in the embryos of honeybee workers relative to drones at 60 hour. (h) Biological functions of up-regulated proteins in the embryos of honeybee drones relative to workers at 60 hour. (i) Biological functions of up-regulated proteins in the embryos of honeybee workers relative to drones at 66 hour. (j) Biological functions of up-regulated proteins in the embryos of honeybee drones relative to workers at 66 hour.

**Figure_S3**


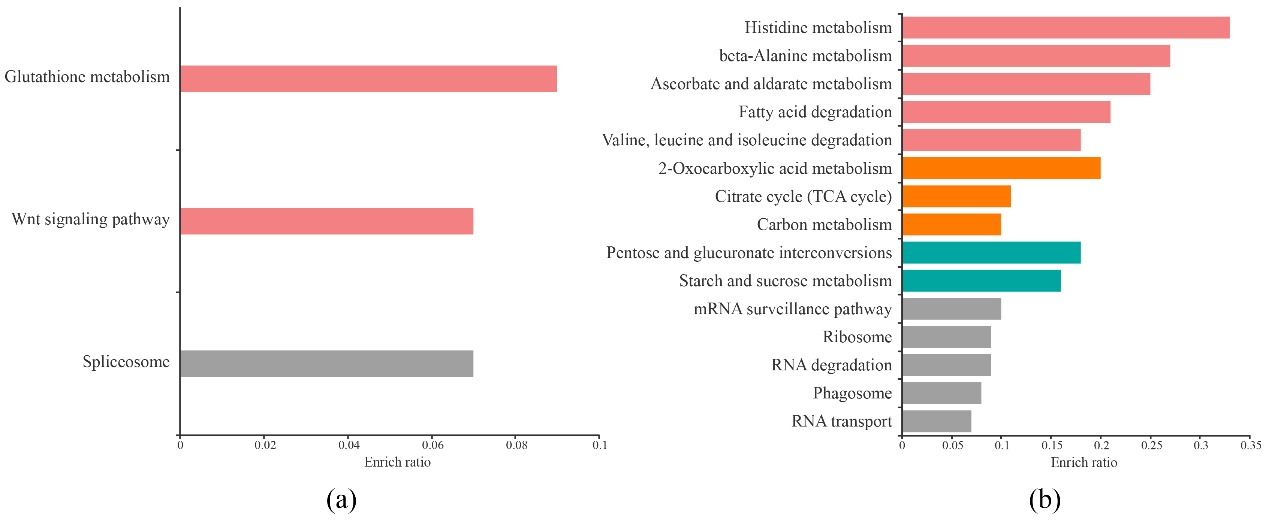


**Figure_S3 Biological pathway enrichment.**

(a) Biological pathway enrichment of up-regulated proteins in the embryos of honeybee workers relative to drones at 60 hour. (b) Biological pathway enrichment of up-regulated proteins in the embryos of honeybee drones relative to workers at 60 hour.
